# On the Tests for Arsenic, in Answer to Mr. Prideaux

**Published:** 1818-10

**Authors:** Joseph Hume


*For the London Medical and Physical Journal.*

### *On the Tests for Arsenic, in Answer to Mr. Prideaux;* by JOSEPH HUME, Esq.

ANOTHER instance of poisoning by arsenic occurred lately, in which the management for detecting the poison devolved entirely upon myself. As I may find it necessary to allude to this case on future occasions, as well as in noticing Mr. Prideaux's last letter, it will be requisite to detail the particulars, the principal part of which are already before the public, having been inserted in some of the London Newspapers, especially in the *Star* of the 23d of July.

On the 22d of May last, a coroner's inquest was held at the King's Arms, Little Woodstock-street, Mary-le-bone, on the body of a young lady, who, from disappointment in love and a consequent state of despondency, had put a period to her life by the means of arsenic. The family at first did not suspect the true cause of her death, although this was preceded by the most violent and agonising symptoms; neither did the medical gentleman, who attended the deceased, consider the signs to have indicated poison as the original cause; on the contrary, he declared the disease to be *cholera morbus.* In justice to this gentleman, however, it is my duty to clear him from the slightest imputation of error in judgment or practical skill; for he never saw the unfortunate victim till within a few minutes before she died, and, on his witnessing some of my experiments, he did not hesitate to give his decided opinion in favour of the verdict returned by the jury. As cholera morbus may become a kind of stalking-horse, I would have this disease narrowly watched with the most jealous attention at all times, and through every step of its progress; for its symptoms are, by some, acknowledged to be very similar to those occasioned by arsenic.

Mr. Holdsworth, of Upper Mary-le-bone-street, another respectable surgeon and a friend of the family, was, I believe, the first person whose suspicions induced an enquiry; these, however, were incited rather by the previous state of the young woman's mind, than the morbid derangements which attended her last moments. This gentleman, by recommendation, brought a small coffee-cup to me, out of which, it was conjectured, the deceased had drunk something of a deleterious nature. As this cup has a handle, and was apparently dry and empty, I considered that, if any arsenic remained in it, I should find it at one side in the bottom; where, from its weight, the poison would naturally subside, while the cup was held in the usual position to the mouth, with the *right* hand. To this part of the vessel I applied a most minute quantity, a mere fractional part of a drop of the ammoniaco-nitrate of silver, conveyed on the point of a small fragment of window-glass. Knowing that this test will instantly detect arsenic in its *dry* state, as well as in solution, I inspected the spot through a magnifying lens, and readily discovered that the test indicated arsenic, and that this proof alone would be sufficient to warrant our proceeding to an inquest. Mr. Holdsworth and I then agreed to the propriety of dissection, and that the contents of the stomach should be obtained to aid the enquiry; accordingly, the operation was performed, and the quantity abstracted amounted to nearly twelve ounces, or three-quarters of a pint, of which Mr. Holdsworth sent to me about an eighth part. The coffee-cup and the small phial of the slimy dark-coloured fluid taken out of the stomach were, therefore, the only sources in this case for the experimental enquiry, which, in the sequel, afforded the most ample and indisputable evidence of the presence of arsenic. The test was cautiously applied to the cup in the first instance, in such quantity as to leave some of the arsenic in reserve for other operations; and, in the present instance, my care was rewarded by success, for a more satisfactory example of evidence from chemical investigation would be altogether unreasonable, unless it were to satisfy the fastidious and ignorant.

I shall briefly relate the method I pursued, conceiving that your readers, particularly your correspondent Mr. Prideaux, will duly appreciate the superior value of tests, and not rely so exclusively on the metallic reproduction of the arsenic, which, I again affirm, is neither so practicable nor decisive.

The coffee-cup contained nearly four ounces of water, or a quarter of a wine-pint, as I afterwards ascertained; and, having no reason to expect any very sensible quantity of arsenic, I began cautiously in this way. About one hundred drops of distilled water were conveyed to that part of the bottom of the cup already described. This was heated slowly by aid of a spirit-lamp till the water began to evaporate, the vessel being gently shaken to promote the solution; the cup with its contents were then removed and allowed to become quite cold. In all cases where *ammoniaco*-nitrate of silver is to be employed, the solution to be examined should be *cold.* A few drops of this solution were then conveyed by means of a piece of glass, from the cup to the surface of a morsel of writing paper, which I find a very convenient receptacle for demonstration. On applying the ammoniaco-nitrate in the usual way, the whole of the solution on the paper assumed the most decided signs of arsenic,—the same fine yellow, and the same flocculent and *curdled* appearance, which so conspicuously distinguishes this precipitate from *phosphate* of silver: the effect was, indeed, so decided and the precipitation so ample, that a saturated solution of arsenic could not have afforded a more satisfactory result.

The same process was followed with ammoniaco-sulphate of copper, in lieu of the silver test, and the evidence was equally conclusive;—*Scheele's green* was the consequence, a precipitate which eminently secures us against the error of confounding arsenic with phosphoric salts. In regard to that fabulous affair—the decoction of onions, I hope to hear no more of this and the other absurdities of a contemporary phalanx.

Having now obtained the true tint and peculiarities of arsenite of silver, and also of arsenite of copper (Scheele's green,) and those from so small a portion of the solution, I did not despair of gaining, from this source alone, enough of the poison to substantiate my evidence of its presence and identity.

The remainder of the solution was then carefully poured out, and one ounce of distilled water put into the cup. This being boiled and allowed to cool as before, gave with the tests exactly the same evident signs of arsenic. During the operation of boiling, the cup was frequently agitated so as to facilitate the solution, and wash down every particle of arsenic that might have attached itself to the sides, even to the edge of the cup. In this way I proceeded, by repeated application of one ounce of water at each boiling, till I had obtained at least eight ounces of solution of arsenic. Even to the last I found some slight indications of this substance, although not so decided as to quantity; for, after the three or four first affusions, there was a regular and rapid diminution.

That portion of the contents of the stomach which I received from Mr. Holdsworth, was allowed to remain undisturbed for at least twelve hours, when it appeared in three distinct strata: the superior stratum was more fluid and nearly transparent; the second had the appearance of dirty coloured water-gruel; and the bottom part was similar to the last, but more viscid in consistence and denser in colour.

The upper stratum was carefully examined, both in its primitive state and when diluted with distilled water, by applying the tests. Neither the silver nor copper test, however, showed the least indication of arsenic. The simple nitrate of silver, indeed, gave an opake cloudy appearance to the fluid, which I ascribed to one of the most prevailing salts in the human system—the muriate of soda.

I pursued the same means of enquiry with the second stratum, but with no better success; I could gather no proof of the existence of arsenic, either before or after dilution, and at last boiling; so that the fair deduction from these experiments is, that there was no arsenic in the state of *solution* in either of these portions of the contents of the stomach,—a fact well worthy the attention of experimentalists.

Having discovered none of the metallic poison in the supernatant portions, I did not hesitate to dilute largely, with cold distilled water, the more condensed residue; well knowing from the very insoluble nature of arsenic, that I should run no risk of losing it by mere affusion. After due subsidence, and before I poured off the water, I examined, through a high magnifier, the sediment, which still remained of a dark slate-colour, and could discern a very few extremely minute white particles, very thinly but not uniformly disseminated through the whole. Here the course I ought next to take was clearly indicated, namely, to persevere, which I did, with repeated affusions and boilings, as had been practised with the coffee-cup, until the whole of the arsenic, if any did exist, should be exhausted and dissolved.

On assaying the first solution, which consisted of only two drams of distilled water, the tests discovered a very strong impregnation of arsenic; even a single drop or two would yield a fair example and proof by either of the tests; in short, it was a strong solution of white oxide of arsenic, and nothing else, as the following process will clearly evince.

The aqueous solution derived from the contents of the stomach, and obtained by repeated boilings upon the sediment, amounted in the whole to about a pint. The solutions from the coffee-cup were also mixed together and added to the other, so that I then possessed about twenty-four ounces of a solution of arsenic. From one-half of this general supply I threw down the arsenic by the ammoniaco-nitrate of silver, and the arsenite of silver thus formed was carefully washed, dried, and put into a proper glass tube, hermetically sealed at one end. That part of the tube only containing the arsenite was exposed to a gentle heat from a spirit lamp, till white fumes began to ascend and attach themselves to the interior and upper part of the tube; and this process was continued, carefully avoiding an increase of heat, more than was barely sufficient for sublimation of the white oxide of arsenic within the bounds of the glass-tube, till I was satisfied that I should find enough for my purpose; I then withdrew the lamp, and suffered the whole to cool. As the sublimation of white oxide of arsenic requires comparatively very little heat, I am astonished that Mr. Prideaux does not attend to this circumstance, for, as he speaks with confidence, I must take it for granted he has a practical knowledge of the subject. But, not to interrupt my process, I shall proceed to say, that I cut off that part of the tube containing the condensed oxide of arsenic; this I placed within a test-tube of somewhat larger diameter, containing distilled water; I then exposed this apparatus to the same lamp, moving and agitating the tube so as to promote solution. Thus I succeeded to regenerate my solution of arsenic, which proved equally susceptible of the tests, and left not a shadow of doubt as to the presence of the same deleterious principle. Is it possible for any phosphate or decoction of onions to interfere with the character of this test? Is there any substance in nature that, under the same circumstances, would *so easily* sublime, and afterwards indicate the same appearances by the same tests so applied? Would any soluble phosphate, even that of ammonia, give the same results? and, I may ask, is not this evidence irresistible?

The unfortunate sufferer, in this case, experienced the same symptoms, and at least in as violent a degree, as the late Mrs. Downing did, whose death gave rise to the trial at Launceston, to which Mr. Prideaux again refers. I am, therefore, more than ever warranted in my opinion in asserting, that in no case whatever can even a strong decoction of onions withstand such excessive and protracted evacuations as took place with these two unfortunate women.

White arsenic, when administered in *substance*, seems to possess a peculiar and insinuating kind of attraction for the villi of the stomach; so much so, that no emetic can dislodge it. On the contrary, when arsenical *solutions* are received into this organ, there is likely to be a greater chance of safety to the individual and more difficulty to the chemist, if he rely upon the *contents of the stomach* only for examination, should the poison have been effectual. Hence it should, I presume, be adopted as a general rule, in all cases where professional evidence is required, and where we are ignorant of the mode employed in administering this poison, to select the very *first* evacuations by vomit, or as early as possible; then to examine the contents of the stomach, should we fail in the first research, and that death has followed, especially if the symptoms have the least similarity to those of cholera morbus. In collecting these contents, we cannot be too sedulous in preserving the crude and more solid matter; every thing mucilaginous or grouty in consistence should be separated and preserved; and the villous membrane carefully divested of every thing of an extraneous nature that can possibly be removed. In a case similar to that I have now detailed, a mere atom of arsenic, the very fraction of a grain, would be a most valuable acquisition for evidence, and would establish the truth most completely, seeing that here no signs of arsenic could be found in the *fluid* portion which we procured from the stomach.

Mr. Prideaux says, that my query, "how could *decoction of onions* remain in the stomach after so much food and medicine had been taken, after so many hours had elapsed, and after such violent retchings, evacuations, and convulsive disturbance of the animal frame?" (for these are my words,) '*is at least as applicable to arsenic.*'

My only observation on this is, that, like all other assertions, his opinion remains to be proved; and the case I have mentioned, which called for my evidence before a coroner's jury, seems to confirm *my* decision on the question, that neither phosphates nor onions should be permitted to interfere as evidence.

If Mr. Prideaux would take the trouble to read what I have already written, he would find, amongst other truths which I have established, that there are many and very obvious advantages to be derived from *ammoniaco*-nitrate of silver. In the first place, when this is presented to a suspected solution of arsenic, the *quantity* of alkali requires no particular caution,—it is already defined; for too much alkali might defeat our purpose. *Simple* nitrate of silver will always at once, and without any alkali, produce a *yellow* precipitate, with all the soluble phosphates that can possibly exist in the human frame; on the contrary, the ammoniaco-nitrate has no such effect, upon phosphate of soda at least; and, had Mr. Prideaux used my test to his saturated solution of phosphate of soda, instead of the "weak solution of lunar caustic which he employed," he would have been convinced that it possessed at least one advantage over other tests. In respect to the question of *lime,* to which Mr. Prideaux alludes, I certainly would not take the *nitrate* of lime. I have already published on this part of the subject, in the Philosophical Magazine; and, in the same work, some years ago,* I strongly recommended nitrate of *lead* as the most effectual test for separating phosphoric *acid;* so that Mr. Prideaux will, I hope, believe me, when I assure him, that nothing can be more easily separated than phosphoric acid from its soluble compounds; and, I may add, that no degree of heat is capable of subliming any phosphate or even phosphoric acid. Hence the very material qualification possessed by *arsenite* of silver when compared with the phosphate of the same metal; the arsenic can be readily disentangled and driven from its base by a very moderate degree of heat, and under the most favourable circumstances, enclosed in glass and exposed during the whole process to the eye of the operator.

I observe a passage in Mr. Prideaux's letter, (p. 188,) where the error seems to have been occasioned by an excess of alkali. Sulphate of copper and subcarbonate of potash were used to detect arsenic, added (*dissolved in,* I suppose) to "the *fluid* of the stomach." I shall here assume that this is a simple solution of arsenic, and go on to say that *ammoniaco*-sulphate of copper, another test strongly advised by me as a collateral supporter, would not produce a blue *precipitate;* for Mr. P. does not say what the "deep blue"

* Philos. Mag. Vol. xx. p. 160.

was. Copper, combined with ammonia, is always *transparent* if in solution. This part of Mr. Prideaux's letter is rather obscure, or else I misconceive his language. Does muriate of *ammonia* exist in the *fluid* of the stomach? Mr. P. adds, that the same phenomena "have occurred in every instance where" he "had an opportunity of trying the experiment."

How can the science of chemistry be considered in a different sense by courts of justice, or rather by a jury, from that of medicine, or any other art or profession? I think the Cornish jury at Launceston, to which, probably, both Mr. Prideaux and I now allude, were just as competent to judge on what may be termed the *physical* part and language of the evidence, as on the *chemical*; and that "common fame" had no influence on their comprehension of the terms, whether *stellated*, *villous*, *cholera morbus*; or *phosphates*, *precipitate*, *acids*, &c. and we may ask, as *diplomas* did so clash and differ in opinion on that occasion, and only one of them had any thing to do with the real examination by *analysis*, what faith can a jury place upon diplomas, certificates, indentures of apprenticeship, or even common fame?

The published trial at Launceston* is now before me; I have long ago read it with attention, and often had occasion to refer to it in conversing with many of my professional friends, who unanimously agree with me in the same opinion. I think there was ample justice done to the decoction of onions; and that the endearing qualities of tenderness, attention, humanity, kindness of heart, and others of the same import, respecting the *character* of the accused, were *duly* considered by the jury.

I am unwilling, for plain reasons, to comment upon the whole of that extraordinary monotony of evidence on *one* side of the question; I may, indeed, be misled by errors of the press, and, therefore, I shall not risk any more of the numerous reflections I have to offer on the subject. If Mr. Prideaux does not consider the following quotation, and the only one I shall make, to be an *erratum*, I shall despair of convincing him of many others of greater import, with which the trial is replete.

The words of a learned sergeant are, addressing himself to a witness; "We have been told, that sulphate of copper, when added to any liquid or fluid containing arsenic, will throw down *a green precipitate?*"—"*Yes*," replied the witness, "*it will have that effect; and I have made that experiment!*" See Trial, p. 117. I quote here only a part of this page; I would, however, recommend the whole, and indeed the entire book itself, to Mr. Prideaux's more mature consideration,

---

* Published at Falmouth, by J. Lake.

before he deems "the experiments used on the Launceston trial" to have been "properly in point."

I shall now close this letter with the expression of the learned judge, who, in his charge to the jury, on the trial of Hussey, at the late assizes at Maidstone, observed, that "*Circumstantial* evidence is sometimes stronger than direct testimony, as circumstances cannot be so *easily* falsified."

Here we have an appropriate motto for the Launceston trial; and, if the mineralogical anecdote, to which Mr. P. alludes (p. 187.), is taken into the account, as it deserves, for it is founded on fact, your readers may then easily collect my opinion of the *chemical* knowledge that was displayed on the occasion. Nothing can be more reprehensible than proclaiming such gross assumptions, such unfounded and delusive doctrine, as, "that nothing short of metallic revival of the arsenic *can bear a man out in swearing to its presence;*" and this, forsooth, is asserted to be the opinion of *all* physicians, chemists, and authorities, both ancient and modern. I have long ago set the whole of these assertions at defiance, and by the most effectual of all modes—by *experiment;* the process now presented to your readers is another specimen of what I wish to inculcate,—that nothing is more readily detected than arsenic; and that its reproduction in the state of *white oxide* is the least liable to deception.

*Long Acre, London;*
*14th Sept.* 1818.

